# Artificial intelligence-enhanced electrocardiography for predicting mortality and major adverse cardiovascular events: A systematic review and narrative synthesis

**DOI:** 10.1371/journal.pone.0360756

**Published:** 2026-10-16

**Authors:** Elhaitham Yasir Awad Musa, Amar Yasir Awad Musa

**Affiliations:** Faculty of Medicine, National Ribat University, Khartoum, Sudan; Azienda Ospedaliero Universitaria Careggi, ITALY

## Abstract

Cardiovascular disease (CVD) remains the leading cause of death worldwide. While clinical scores provide modest predictive accuracy, the electrocardiogram (ECG) is rarely leveraged for prognosis. Artificial intelligence (AI) and deep learning now enable extraction of high-dimensional features from ECG waveforms for predicting cardiovascular outcomes. Our objective was to systematically evaluate the prognostic performance of AI-enabled ECG models for predicting all-cause mortality, cardiovascular mortality, and major adverse cardiovascular events (MACE). We searched PubMed, Cochrane CENTRAL, IEEE Xplore, and Google Scholar through November 2025 for studies using AI/ML models with 12-lead or single-lead ECGs to predict all-cause mortality, cardiovascular mortality, or MACE in adults (≥18 years old). Eligible studies included adults (≥18 years) using ECG as primary input. Two reviewers independently screened and extracted data. Narrative synthesis was conducted due to heterogeneity. Thirteen studies were included (~5.9 million patients). Eleven reported all-cause mortality (8 short-term: 30-day to 1-year), four reported cardiovascular mortality, and eight assessed MACE (4 composite MACE, 4 individual components). Models predominantly used convolutional neural networks [CNNs] on raw ECG waveforms. C-statistics ranged 0.70–0.89 for mortality prediction. Definitions and follow-up durations varied, limiting direct comparison. AI-ECG models demonstrate strong potential for predicting mortality and cardiovascular events with consistent performance and scalability. However, prospective validation and standardization are essential before clinical implementation.

## 1. Introduction

Cardiovascular disease (CVD) remains the leading global cause of death [1]. Early risk identification is critical for preventive strategies [2]. Clinical prediction tools provide modest performance (AUCs ~ 0.65–0.75) [2,3] and often miss subclinical patterns indicating elevated risk.

The 12-lead electrocardiogram (ECG) is a non-invasive, widely accessible tool in conventional medical practice. While consumer single-lead wearables are gaining popularity, the vast majority of validated prognostic data relies on standard clinical 12-lead recordings [4].

Artificial intelligence (AI), particularly deep learning, has emerged as a transformative approach for ECG analysis. AI models can extract latent features from raw waveforms and predict clinical outcomes without explicit feature engineering [5–8]. Unlike conventional ECG interpretation, which relies on pre-specified morphological criteria, deep learning models process raw voltage-time data across all 12 leads simultaneously, enabling detection of subtle prognostic patterns that are invisible to human readers. Several studies suggest AI-enhanced ECG models can predict cardiovascular mortality and major adverse cardiovascular events (MACE) [9–12].

However, no systematic synthesis has yet evaluated AI-ECG models specifically for mortality or MACE prediction. The evidence base has grown substantially over the past decade, spanning diverse populations, clinical settings, model architectures, and follow-up horizons — yet the heterogeneity in outcome definitions, validation approaches, and performance metrics makes cross-study interpretation difficult without structured synthesis. As AI-ECG tools approach clinical readiness, a rigorous evaluation of their prognostic performance and methodological quality is urgently needed to inform both clinical practice and future research priorities.

In this review, we systematically evaluate the prognostic performance of AI-ECG models for predicting all-cause mortality, cardiovascular mortality, and MACE in adults, assess methodological quality, and identify priorities for future clinical validation and implementation.

## 2. Methods

### 2.1 Protocol and registration

This systematic review was conducted in accordance with the Preferred Reporting Items for Systematic Reviews and Meta-Analyses (PRISMA) 2020 guidelines [13] and was prospectively registered with the PROSPERO database (Registration ID: CRD420251244907). Full Details in S1 Appendix.

### 2.2 Eligibility criteria

We included studies using AI/ML models with surface ECGs (12-lead or single-lead) as the primary input to predict all-cause mortality, cardiovascular mortality, or MACE in adults (≥18 years) in any clinical or community setting. Both internally and externally validated models were eligible, across retrospective and prospective cohort designs, model development and validation studies, and randomized controlled trials (RCTs).

Studies employing a two-step design — wherein AI models first identify intermediate ECG phenotypes subsequently used to stratify mortality or MACE risk — were also eligible, provided the ECG remained the primary model input.

We excluded pediatric-only studies, ECG-age or heart-age models, diagnostic-only models without prognostic intent, models not using ECG as a primary input, and conference abstracts without extractable outcome data.

Following data extraction, individual MACE components (myocardial infarction [MI], stroke, heart failure [HF], ventricular arrhythmia) were additionally captured where reported by included studies, as these outcomes were consistently present and provided important prognostic context. Outcome definitions were extracted exactly as defined and reported by each study’s authors. This included extracting specific composite sub-endpoints, such as atherosclerotic cardiovascular disease (ASCVD), because its standard clinical components (e.g., non-fatal myocardial infarction, stroke, and cardiovascular death) directly overlap with classic MACE definitions, making it highly relevant to our prognostic scope.

### 2.3 Information sources

We systematically searched four electronic databases: PubMed/MEDLINE, Cochrane CENTRAL, IEEE Xplore, and Google Scholar, from inception through November 25, 2025, with no date restrictions applied. All searches were limited to English-language publications. Google Scholar results were manually screened due to the platform’s limited advanced search capabilities, and the search was capped at the first 200 results. This aligns with established grey literature methodologies, as screening beyond this threshold introduces a high noise-to-signal ratio with exponentially diminishing scientific returns [14]. Conference abstracts were additionally screened where identified through the searches.

### 2.4 Search strategy

Search strings were constructed using three concept blocks combined with Boolean operators: ECG-related terms (“electrocardiogram,” “ECG,” “EKG”), AI and machine learning terms (“artificial intelligence,” “machine learning,” “deep learning,” “neural network”), and prognostic outcome terms (“mortality,” “cardiovascular death,” “MACE,” “major adverse cardiovascular events”). Exclusion terms (“heart age,” “ECG age”) were applied across all databases to remove ECG-age estimation studies. Field-specific syntax was applied where supported — PubMed searches used [Title/Abstract] field tags, IEEE Xplore used metadata-only searching across title, abstract, and index terms, and Cochrane CENTRAL searches defaulted to title, abstract, and keyword fields. Full database-specific search strings are provided in S1 Appendix.

### 2.5 Study selection

Two independent reviewers screened all records by title and abstract against the eligibility criteria, followed by full-text review of potentially eligible studies. Disagreements at either stage were resolved by discussion and consensus. A PRISMA 2020 flow diagram detailing the screening process is provided in Fig 1.

**Fig 1:**
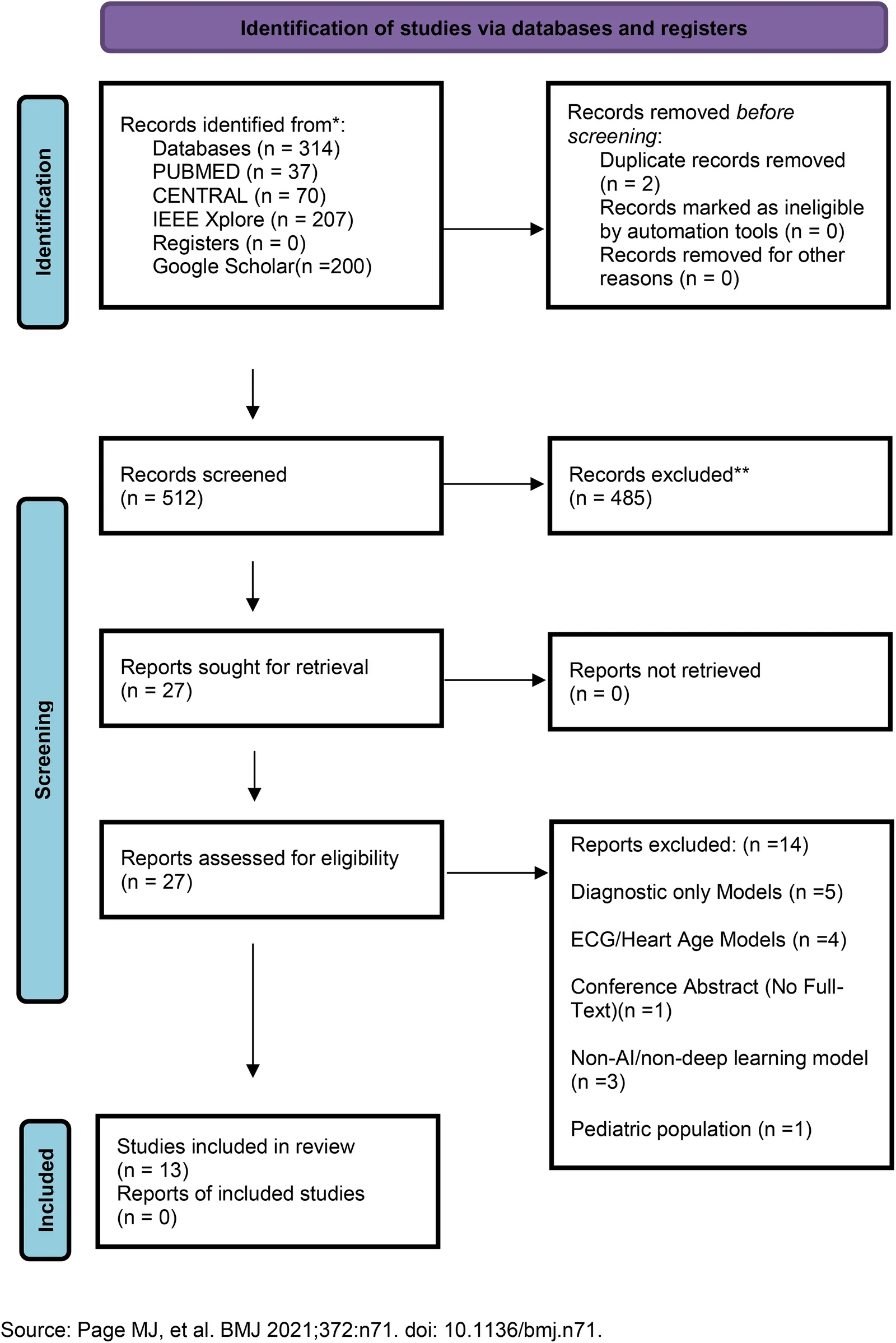
PRISMA 2020 flow diagram.

### 2.6 Data extraction

Data were extracted independently by two reviewers using a standardised extraction form. Extracted fields included: study design, country and clinical setting, sample size, ECG input type, AI model architecture, training and validation approach, outcomes reported, follow-up duration, and performance metrics (AUC, C-index, HR with 95% CI). Discrepancies were resolved by consensus.

### 2.7 Synthesis of results

Statistical pooling and subgroup meta-analyses (e.g., for 1-year all-cause mortality) were deemed inappropriate a priori due to intractable clinical, methodological, and statistical heterogeneity. Even within specific temporal endpoints, baseline populations varied drastically, ranging from general unselected hospital cohorts to highly specific disease populations. Furthermore, the reported statistical metrics were mutually incompatible for quantitative synthesis, mixing standard binary classification metrics (AUC) with time-to-event survival metrics (C-index). Critically, several large-scale studies utilized overlapping regional datasets; pooling these datasets would introduce severe bias through the double-counting of patients. According to the Cochrane Handbook for Systematic Reviews of Interventions [15], attempting statistical pooling or calculating statistical heterogeneity metrics (such as I-squared) under these conditions violates core assumptions of baseline homogeneity and yields mathematically invalid summary estimates. Therefore, a rigorous narrative synthesis was performed. Risk of bias was assessed for each included study using PROBAST [16], with detailed rationale provided in S1 Appendix.

## 3. Results

### 3.1 Study selection

Out of 512 records screened, 27 full-text articles were reviewed in detail. A total of 13 studies met inclusion criteria and were included in the final synthesis. Fourteen studies were excluded at full-text review: eleven due to incompatible focus or format (ECG-age estimation, diagnostic-only outcomes, pediatric population, or conference abstracts lacking full text), and three due to the use of traditional statistical regression models without neural network architecture (non-AI/ML model). A PRISMA flow diagram is provided in Fig 1.

### 3.2 Overview of included studies

The included studies were published between 2016 and 2025 and originated from diverse settings including the United States, Canada, Taiwan, Switzerland, South Korea, the United Kingdom, and Brazil. All studies used either 12-lead or single-lead surface ECGs as model inputs, with sample sizes ranging from several hundred to over two million ECGs. Twelve studies employed deep learning architectures (typically convolutional neural networks); one study systematically compared a ResNet-based deep learning model against an XGBoost approach [10].

Across the included literature:

Eleven studies reported all-cause mortality, with eight evaluating short-term mortality (≤1 year), and five assessing long-term mortality (>1 year or extended follow-up), with some overlap between these categories. Four studies reported cardiovascular mortality. Four studies evaluated composite MACE outcomes, and an additional four reported on individual MACE components (e.g., MI, HF hospitalization, stroke) without composite MACE endpoints.

Summary characteristics of the included studies are presented in Table 1

**Table 1:** Characteristics of included studies.

Study	Country	Sample Size	ECG Input	Model Type	Predicted Outcome(s)	Validation Type
Raghunath et al.	USA	2,338,833 ECGs (536,661 patients)	12-lead (raw)	Deep CNN	All-cause mortality	Internal
Lin et al. (ECG-Surv)	Taiwan	4.9M ECGs (1,684,298 pts)	12-lead (raw)	Deep CNN	All-cause, CV mortality	Both
Lin et al. (ECG-MACE)	Taiwan	2.8M ECGs (1,078,629 pts)	12-lead (raw)	Multitask DL	MACE components, all-cause mortality	Both
Hughes et al.	USA	1 ECG/536,449 pts	12-lead (raw)	Deep CNN	CV mortality, MACE components	Both
Ouyang et al.	USA	112,794 ECGs (45,969 pts)	12-lead (raw)	Deep CNN	30-day mortality, MACE	Both
Champetier et al.	Switzerland	6,098 pts	12-lead (digital)	PreOpNet (CNN)	MACE, mortality	External
Sau et al.	US	1,163,401 ECGs (189,539 pts)	8-lead (raw)*	Deep CNN	All-cause mortality, CV mortality, MACE components	Both
Lee et al.	South Korea	16,228 ECGs (3,894 HFrEF pts)	12-lead (raw)	Deep CNN	All-cause mortality	Internal
Kanaji et al.	USA	1 ECG/305 pts	12-lead (raw)	Multiple CNN tools	MACE	Internal
Sun et al.	Canada	1,605,268 ECGs (244,077 pts)	12-lead (raw)	ResNet (Deep CNN)	Mortality	Internal
Lu et al.	Brazil	2.3M ECGs (1,558,772 patients)	12-lead (raw)	Deep CNN	All-cause mortality	Internal
Alrumayh et al.	UK	1 ECG/1,007 pts	Single-lead (raw)	CNN (low EF)	MACE, mortality	Internal
Tsai et al.	Taiwan	512,805 ECGs (271,407 pts)	12-lead (raw)	CNN	All-cause mortality	Both

*used 8 leads (I, II, V1–V6) from a standard 12-lead recording; excluded leads are linear combinations of retained leads and do not represent a distinct input modality.

Abbreviations: AMI = Acute Myocardial Infarction; AUC = Area Under the Curve; CNN = Convolutional Neural Network; CV = Cardiovascular; DL = Deep Learning; ECG = Electrocardiogram; HFrEF = Heart Failure with reduced Ejection Fraction; MACE = Major Adverse Cardiovascular Events; NR = Not Reported; pts = patients.

Complete all-cause mortality data for all 11 studies are provided in S1 Table. and Complete cardiovascular mortality data are provided in S2 Table and Complete MACE data are presented in S3 Table.

### 3.3 All-cause mortality prediction

Eleven studies reported all-cause mortality outcomes, spanning both short-term (30-day to 1-year) and long-term (>1-year) horizons. Short-Term All-Cause Mortality was reported by eight studies, Long-Term All cause mortality by five.

#### Short-term all-cause mortality (n = 8).

Eight studies evaluated AI-ECG models for predicting short-term all-cause mortality (30-day to 1-year):

**Sun et al.** developed a ResNet-based deep learning model using over 1.6 million ECGs to predict short-term mortality at 30 days and 1 year, evaluated on an internal holdout dataset. In the validation cohort, the ECG-only model achieved an AUC of 0.843 (95% CI 0.838–0.848) for 30-day mortality and 0.812 (95% CI 0.808–0.816) for 1-year mortality [10].

**Tsai et al.** employed a CNN trained on a Taiwanese hospital cohort (Tri-Service General Hospital) ECG database to predict 1-year all-cause mortality. The model demonstrated strong performance, with an AUROC of 0.894 (95% CI 0.879–0.909) [17].

**Raghunath et al.** developed a deep learning model using a total cohort of 2,338,833 ECGs from Geisinger. In the total holdout test set, the model predicted 1-year all-cause mortality with an overall AUC of 0.88 and a hazard ratio of 8.1 (95% CI 7.9–8.2). Notably, the model maintained high performance even in a subgroup of patients with ‘Normal’ ECG interpretations (AUC 0.85) *and a hazard ratio of 9.5 (95% CI 9.0–10.0)*, highlighting its ability to detect subclinical prognostic signals [9].

**Ouyang et al.** developed PreOpNet, a deep learning model for postprocedural risk stratification in patients undergoing inpatient procedures. In a held-out test set of 4,581 patients, the model predicted 30-day all-cause mortality with an AUC of 0.83 (95% CI 0.79–0.87), and was subsequently validated across two external institutions [18].

**Champetier et al.** externally validated PreOpNet, an ECG-based model for 30-day mortality in high-risk surgical patients, with an AUC of 0.707 (95% CI 0.672–0.742) [19].

**Lin et al. (ECG-MACE)** reported 1-year all-cause mortality predicted by their multitask deep learning model ECG-MACE, developed using 2.8 million ECGs from Taiwan’s CGMH hospital network. The model achieved high performance for this endpoint, with an AUC of 0.89 for 1-year all-cause mortality prediction, the ECG-MACE model also achieved an external AUROC of 0.83 for 1-year all-cause mortality at an independent institution, and demonstrated long-term prognostic value in internal analyses up to 10 years [12].

**Lin et al. (ECG-surv)** developed a ResNet-18 model trained on 4.9 million ECGs, achieving a c-index of 0.860 for 1-year mortality [20].

**Lee et al.** developed a deep-learning model to predict 1-year all-cause mortality in heart failure with reduced ejection fraction (HFrEF) patients using ECGs. In the test set, the model achieved an AUC of 0.826 (95% CI 0.794–0.859), and the deep learning algorithm was an independent predictor with an adjusted HR of 4.12 (95% CI 2.32–7.33) [21].

#### Long-term all-cause mortality (n = 5).

Five studies assessed long-term outcomes (>1 year):

**Sun et al.** was also evaluated for 5-year all-cause mortality. The model achieved an AUC of 0.798 (95% CI 0.792–0.803) in the validation cohort, maintaining consistent discrimination across time [10].

**Alrumayh et al.** applied a single-lead AI model to detect low left ventricular ejection fraction (LVEF); patients flagged as high-risk had a significantly increased 2-year all-cause mortality (HR 1.56, 95% CI 1.06–2.29) [22].

**Sau et al.** developed a deep learning model using BIDMC, Boston, USA ECGs to predict long-term cardiovascular outcomes, including all-cause mortality. In a cohort of 189,539 patients with a mean follow-up of 5.46 years, the model achieved a concordance index (c-index) of 0.775 for predicting all-cause death. The AI-ECG model outperformed traditional clinical risk scores and demonstrated significant prognostic value even in patients with normal conventional ECG interpretations [23].

**Tsai et al.** employed a CNN trained on a Taiwanese hospital cohort to predict 10-year all-cause mortality. The model demonstrated strong performance, with a C-index of 0.858 in internal validation and 0.836 in external validation and an adjusted HR of 14.16 (adjusted for age and sex) (95% CI 11.33–17.70) for high-risk versus low-risk individuals [17].

**Lu et al.** developed an interpretable deep learning model that identified cardiac abnormalities from 2.3 million ECGs from a Brazilian primary care network, which were then used to stratify mortality risk over up to 7 years. Using ECG-predicted results, subjects with sinus tachycardia and atrial fibrillation demonstrated the highest adjusted mortality risk, with HRs of 2.24 (95% CI 1.96–2.57) and 2.22 (95% CI 1.99–2.48) respectively [24].

These findings support the use of AI-ECG as a robust prognostic tool for mortality risk stratification, with performance often exceeding traditional clinical scores across various timeframes and populations.

### 3.4 Cardiovascular mortality prediction

Four studies predicted Cardiovascular Mortality outcomes:

**Hughes et al.** developed the SEER model to predict 5-year cardiovascular mortality, with an internal AUC of 0.83 and external validation AUCs of 0.78 and 0.83 [11].

**Lin et al. (ECG-surv)** developed a deep learning model based on a ResNet-18 architecture to predict 1-year cardiovascular mortality using 4.9 million 12-lead ECGs from Chang Gung Memorial Hospital (CGMH), Taiwan. The model was trained with a time-to-event survival framework and achieved a c-index of 0.891 for cardiovascular death, This study represents one of the largest datasets used in ECG-AI modeling and demonstrated excellent prognostic discrimination for CV mortality at 1 year [20].

**Sau et al.** developed an ECG-based AI model using 1,163,401 ECGs from Beth Israel Deaconess Medical Center (BIDMC), Boston, USA to predict major adverse cardiovascular outcomes, including cardiovascular mortality. In this study, the model achieved a C-index of 0.832 (95% CI 0.831–0.834) for cardiovascular death over a mean follow-up of 5.46 years [23].

**Tsai et al.** developed a survival deep learning model using 512,805 ECGs from 271,407 patients across two Taiwanese hospital cohorts to predict long-term cardiovascular mortality. The high-risk group identified by the model had a significantly elevated cardiovascular mortality risk compared to the low-risk group, with an HR of 18.50 (95% CI 9.82–34.84) in internal validation and 13.74 (95% CI 8.57–22.04) in external validation. This study highlights the long-term predictive capacity of AI-ECG models and demonstrates applicability across extended follow-up horizons [17].

These studies support the prognostic use of ECGs beyond diagnostic boundaries and demonstrate that AI can detect latent features related to future risk, even in asymptomatic individuals.

### 3.5 Major adverse cardiovascular events (MACE) composite

Four studies evaluated AI-ECG models for predicting composite MACE.

**Ouyang et al.** developed PreOpNet, a deep learning model using preoperative 12-lead ECGs to predict adverse outcomes across inpatient and outpatient procedural settings. The composite MACE outcome included mortality, cardiac arrest, myocardial infarction, heart block, and pulmonary oedema. In the internal test set, the model achieved an AUC of 0.77 (95% CI 0.73–0.80) for 30-day MACE [18].

Subsequently, **Champetier et al.** externally validated PreOpNet. In a cohort of 6,098 high-risk surgical patients from University Hospital Basel, Switzerland, the model achieved an AUC of 0.675 (95% CI 0.649–0.701) for 30-day MACE and 0.707 (95% CI 0.672–0.742) for 30-day mortality, with incremental prognostic value demonstrated when combined with clinical and biomarker data [19].

**Kanaji et al.** focused on Takotsubo cardiomyopathy patients, a group typically considered lower-risk, and applied multiple AI-ECG algorithms to detect high-risk patterns at baseline. Among 305 patients, composite MACE — defined to include Takotsubo recurrence alongside cardiovascular death, MI, HF hospitalization, and stroke — occurred in 26.6% over a median follow-up of 4.8 years. In multivariable analysis, the presence of one high-risk AI-ECG feature was independently associated with MACE (HR 2.38, 95% CI 1.01–5.61), with risk escalating further with two or more features (HR 4.42, 95% CI 1.83–10.7), demonstrating a graded prognostic relationship in a population where conventional risk tools are lacking [25].

**Alrumayh et al.** showed that an AI-ECG model predicting low EF uncovered risk even in patients with normal echocardiographic function. Among patients with LVEF ≥50%, those who screened positive with AI-ECG had significantly higher 2-year MACE rates. In the broader cohort, an AI-predicted LVEF ≤40% was associated with an adjusted HR of 1.93 (95% CI 1.39–2.69) for MACE, suggesting the model detected subtle abnormalities not evident on imaging [22].

### 3.6 MACE components

Four studies reported standalone adverse cardiovascular events without a composite MACE outcome:

**Hughes et al.** developed the SEER model, a deep learning-derived ECG risk score that demonstrated significant prognostic discrimination for individual cardiovascular outcomes. In the top tertile of SEER risk, age- and sex-adjusted hazard ratios were 3.2 (2.9–3.5) for heart failure, 2.0 (1.7–2.5) for myocardial infarction, 1.6 (1.4–1.9) for stroke, and 4.2 (3.0–5.7) for sudden cardiac death [11].

**Sau et al.** developed and validated an AI-ECG model that predicted ASCVD and heart failure events over a mean follow-up of 5.46 years. The model achieved an internal C-index of 0.696 for ASCVD prediction (0.643 in the external UK Biobank cohort) and 0.787 for heart failure (0.768 externally), outperforming traditional risk scores across cohorts [23].

**Tsai et al.** applied their survival deep learning model to a Taiwanese hospital cohort to assess long-term incidence of heart failure, myocardial infarction, and stroke. The high-risk group demonstrated significantly elevated HRs for new-onset HF (HR 6.66, 95% CI 4.54–9.77), AMI (HR 4.01, 95% CI 2.24–7.17), and stroke (HR 2.15, 95% CI 1.70–2.72), with results validated in an independent external cohort [17].

**Lin et al.** ECG-MACE (2025) developed a multi-task deep learning model (ECG-MACE) using over 2.8 million ECGs from a Taiwanese hospital network to simultaneously predict four 1-year outcomes: heart failure hospitalization, myocardial infarction, ischemic stroke, and all-cause mortality. The model achieved strong performance—AUCs of 0.90 for heart failure, 0.85 for myocardial infarction, 0.76 for stroke, and 0.89 for all-cause mortality. Importantly, the model demonstrated long-term prognostic value: individuals flagged positive for future heart failure had a 15-fold higher incidence of the event over 10 years, while those flagged for myocardial infarction had an 8-fold higher incidence. The model also outperformed the Framingham Risk Score in predicting 5-year composite MACE, emphasizing the value of ECG-based risk stratification in primary prevention [12].

A comprehensive summary of the prognostic performance metrics for all included models—spanning short- and long-term all-cause mortality, cardiovascular mortality, and MACE components—is presented in Table 2.

**Table 2:** Key prognostic performance metrics.

Study (Year)	Outcome	Follow-up	Sample Size	Internal AUC/ C-index	HR (95% CI)	Validation
SHORT-TERM ALL-CAUSE MORTALITY						
Sun (2023)	mortality	30 days	1.6M ECGs	0.84	NR	Internal
Sun (2023)	mortality	1 year	1.6M ECGs	0.81	NR	Internal
Raghunath (2020)	mortality	1 year	168,914 patients	0.88	8.1 (7.9 −8.2)	Internal
Lin ECG-MACE (2025)	mortality	1 year	2.8M ECGs	0.89/ 0.83	NR	Both
Lin ECG-surv (2025)	mortality	1 year	4.9M ECGs	0.860/ 0.813	NR	Both
Tsai (2023)	mortality	1 year	512K ECGs	0.894/ 0.858	NR	Both
Ouyang (2024)	mortality	30 days	4,581 patients	0.83/ (0.75-0.79)	NR	Both*
Champetier (2025)	mortality	30 days	6,098 pts	NR/ 0.707	NR	External
Lee (2025)	mortality	1 year	3,894 pts	0.826	4.12 (2.32–7.33)	Internal
LONG-TERM ALL-CAUSE MORTALITY						
Tsai (2023)	mortality	10 years	512K ECGs	0.86/ 0.84	14.16 (11.33-17.70)/ 10.59 (8.32–13.48)	Both
Sau (2024)	Continuous survival	Mean 5.46 yr	1.2M ECGs	0.78/ 0.638 (UKB); Range: 0.638–0.773	NR	Both
Alrumayh (2025)	mortality	2 years	1,007 pts	0.75	1.56 (1.06–2.29)	Internal
Sun (2023)**	mortality	5 years	1.6M ECGs	0.80	NR	Internal
Lu (2024)	mortality	7 years	2.3M ECGs	NR	ST: 2.24 (1.96–2.57); AF: 2.22 (1.99–2.48)	Internal
CARDIOVASCULAR MORTALITY						
Hughes (2023)	CV death	5 years	536K pts	0.83/ (0.78–0.83)	NR	Both
Lin ECG-surv (2025)	CV death	1 year	4.9M ECGs	0.89/ NR	NR	Both
Sau (2024)	CV death	Mean 5.46 yr	1.2M ECGs	0.83/ 0.70	NR	Both
Tsai (2023)	CV mortality	10 years	512K ECGs	NR	18.50 (9.82–34.84)/ 13.74 (8.57–22.04)	Internal
COMPOSITE MACE						
Ouyang (2024)	MACE	30 days	4,581 patients	0.77	NR	Internal
Kanaji (2024)	MACE	Median 4.8 yr	305 pts	NR	4.42 (1.83-10.66)	Internal
Alrumayh (2025)	MACE	2 years	1,007 pts	0.75	1.93 (1.39–2.69)	Internal
Champetier (2025)	MACE	30 days	6,098 pts	NR/ 0.68	NR	External
MACE COMPONENTS						
Lin ECG-MACE (2025)	HF hospitalization	1 year	2.8M ECGs	0.90	NR	Internal
Lin ECG-MACE (2025)	AMI	1 year	2.8M ECGs	0.85	NR	Internal
Lin ECG-MACE (2025)	Stroke	1 year	2.8M ECGs	0.76	NR	Internal
Tsai (2023)	AMI	10 years	512K ECGs	NR	4.01 (2.24–7.17)	Internal
Tsai (2023)	Heart failure	10 years	512K ECGs	NR	6.66 (4.54–9.77)	Internal
Tsai (2023)	Stroke	10 years	512K ECGs	NR	2.15 (1.70–2.72)	Internal
Sau (2024)	ASCVD	Mean 5.46 yr	1.2M ECGs	0.70/ 0.64	NR	Both*
Sau (2024)	Heart failure	Mean 5.46 yr	1.2M ECGs	0.79/ 0.77	NR	Both*
Sau (2024)	Ventricular arrhythmia	Mean 5.46 yr	1.2M ECGs	0.76/ 0.72	NR	Both*
Hughes (2023)	Heart failure Hospitalization	5 years	536K pts	0.76/ NR	3.2 (2.9–3.5)	Internal
Hughes (2023)	AMI	5 years	536K pts	NR	2.0 (1.7–2.5)	Internal
Hughes (2023)	Stroke	5 years	536K pts	NR	1.6 (1.4–1.9)	Internal
Hughes (2023)	SCD	5 years	536K pts	NR	4.2 (3.0–5.7)	Internal
Hughes (2023)	ASCVD	5 years	536K pts	0.67/ 0.63	2.4 (2.2–2.7)	Both

Abbreviations: AMI, acute myocardial infarction; ASCVD, atherosclerotic cardiovascular disease; AUC, area under the curve; CI, confidence interval; CV, cardiovascular; ECG, electrocardiogram; Ext, external validation; HF, heart failure; HR, hazard ratio; Int, internal validation; MACE, major adverse cardiovascular events; NR, not reported; pts, patients; SCD, sudden cardiac death.

Hazard ratios represent high-risk vs. comparison groups as defined by each study’s AI-ECG model. MACE definitions varied by study. ASCVD as reported by Sau et al. [23] represents a composite sub-endpoint of atherosclerotic cardiovascular events rather than a discrete component. Note: 95% Confidence Intervals for all AUC and C-index values are detailed in S1–S3 Tables. Hazard ratios are presented with 95% CIs where applicable.

*Where studies utilized multiple distinct external validation cohorts, the range of external metrics is presented. Ouyang et al. [18] cohorts: CUMC (AUC 0.79), SHC (AUC 0.75). Hughes et al. [11] cohorts: Columbia (AUC 0.83), Cedars-Sinai (AUC 0.78). Sau et al. [23] tested across 4 external cohorts (range 0.64–0.77); the lowest bound (0.64) reflects the UK Biobank cohort.

**The 5-year mortality evaluation in Sun et al. [10] (2023) used a specific sub-cohort of n = 55,650.

While the performance metrics in Table 2 highlight the predictive strength of these AI-ECG models, the specific clinical endpoints evaluated varied considerably across the literature. To clarify this heterogeneity, Table 3 provides a structured overview mapping exactly which mortality and cardiovascular outcomes were reported by each included study.

**Table 3:** Summary of outcomes reported by included studies.

Study	All-Cause Mortality	CV Mortality	Composite MACE	MACE Components
Raghunath (2020)	● (1y)	—	—	—
Sun (2023)	● (30d, 1y, 5y)	—	—	—
Lin ECG-surv (2025)	● (1y)	● (1y)	—	—
Lin ECG-MACE (2025)	● (1y)	—	—	● (HF Hospitalization, MI, stroke)
Hughes (2023)	—	● (5y)	—	● (AMI, Stroke, HF, SCD, ASCVD)
Ouyang (2024)	● (30d)	—	● (30d)	—
Champetier (2025)	● (30d)	—	● (30d)	—
Sau (2024)	● (long-term)	● (long-term)	—	● (ASCVD, HF, VA)
Lee (2025)	● (1y)	—	—	—
Kanaji (2024)	—	—	● (5y)	—
Lu (2024)	● (7y)	—	—	—
Alrumayh (2025)	● (2y)	—	● (2y)	—
Tsai (2023)	● (1y, 10y)	● (10y)	—	● (AMI, HF, stroke)
Total Studies	11	4	4	4

Legend: ● Outcome reported; — Outcome not reported. Timepoints in parentheses indicate follow-up duration.

Abbreviations: AMI, acute myocardial infarction; CV, cardiovascular; d, day; HF, heart failure; MACE, major adverse cardiovascular events; MI, myocardial infarction; SCD, sudden cardiac death; y, year.

### 3.7 Risk of bias assessment

The overall methodological quality and risk of bias for the included literature, as assessed via the PROBAST tool, are summarized in Table 4.

**Table 4:** Summary of PROBAST risk of bias and applicability assessment.

Study	Participants	Predictors	Outcome	Analysis	Overall RoB	Applicability
Raghunath (2020)	Low	Low	Low	Low	Low	Unclear
Sun (2023)	Low	Low	Low	Low	Low	Low
Lin ECG-surv (2025)	Low	Low	Low	Low	Low	Low
Lin ECG-MACE (2025)	Low	Low	Low	Low	Low	Low
Hughes (2023)	Low	Low	Low	Low	Low	Low
Ouyang (2024)	Unclear	Low	Low	Low	Unclear	Unclear
Champetier (2025)	Low	Low	Low	Low	Low	Unclear
Sau (2024)	Low	Low	Low	Low	Low	Low
Lee (2025)	Low	Low	Low	Unclear	Unclear	Unclear
Kanaji (2024)	Unclear	Low	Low	High	High	High
Lu (2024)	Low	Low	Low	Unclear	Unclear	Low
Alrumayh (2025)	Unclear	Low	Low	High	High	Unclear
Tsai (2023)	Low	Low	Low	Low	Low	Low

RoB = Risk of Bias. Low risk indicates all quality criteria met; High risk indicates significant methodological concerns; Unclear risk indicates insufficient information to make judgment.

## 4. Discussion

This systematic review evaluated 13 studies examining the prognostic utility of artificial intelligence-enabled electrocardiograms (AI-ECGs) for predicting cardiovascular outcomes in adults. Collectively, the evidence demonstrates that deep learning models trained on raw ECG waveforms can predict all-cause mortality, cardiovascular mortality, and MACE with performance that frequently exceeds conventional risk stratification tools across diverse clinical settings, populations, and time horizons.

Short-term mortality discrimination was strong across eight studies, with AUCs spanning 0.707 to 0.894. Notably, this range reflects setting heterogeneity rather than model inconsistency: the lower bound comes from 30-day perioperative mortality prediction in surgical patients, while the upper bound reflects 1-year prediction in a general hospital population. That AI-ECG performs meaningfully across both contexts of acute operative risk and chronic cardiovascular prognosis speaks to the generalisability of the prognostic signal embedded in the raw waveform.

Foundational work by Raghunath et al. [9] demonstrated on a massive scale (over 2.3 million ECGs) that these models can accurately predict 1-year mortality even when the ECG is interpreted as completely ‘normal’ by attending physicians. This explicitly proves that the algorithms identify complex non-linear patterns that standard clinical reading protocols are not designed to quantify.

Long-term prediction proved equally feasible, with models achieving discrimination over 5- and 10-year horizons. The use of survival neural network architectures which model time-to-event rather than fixed binary endpoints represents a methodological advance over earlier classification approaches, enabling richer risk trajectories and more appropriate handling of censored data.

Sau et al. [23] similarly demonstrated the breadth of this approach, achieving a c-index of 0.775 for all-cause mortality in a BIDMC secondary care cohort and maintaining discrimination across four transnational external validation cohorts — a degree of geographic generalisability rarely achieved in AI-ECG research. The extension of this evidence to a Brazilian primary care network of 2.3 million ECGs further demonstrates that the prognostic signal generalises beyond high-income academic settings.

For MACE, the findings carry particular clinical significance. The ability of a preoperative ECG alone to predict 30-day adverse events in surgical populations suggests a low-cost alternative or supplement to existing perioperative risk calculators. More striking is the finding that AI-ECG identified elevated MACE risk in patients with preserved ejection fraction on echocardiography — indicating that the model detects subclinical myocardial vulnerability that standard imaging misses. The successful simultaneous prediction of four distinct cardiovascular endpoints by a single multitask model further suggests that the ECG encodes far richer prognostic information than its conventional interpretation reveals.

Individual component prediction — incident heart failure, myocardial infarction, stroke, and sudden cardiac death — was demonstrated across four studies with discrimination and hazard ratios consistently exceeding conventional risk tools. Hughes et al. [11] reported age- and sex-adjusted HRs of 4.2 for sudden cardiac death and 3.2 for heart failure hospitalisation in the top risk tertile, while Tsai et al. [17] demonstrated HRs of 6.66 for new-onset heart failure and 4.01 for myocardial infarction over a 10-year horizon. These findings reinforce the broader argument that AI-ECG adds clinically actionable prognostic information beyond what conventional ECG interpretation currently extracts — and that this information spans multiple outcome domains simultaneously.

Furthermore, while these models demonstrate robust predictive capacity, their clinical interpretation must account for underlying baseline cardiometabolic risk factors, which remain significant clinical confounders that can independently drive MACE trajectories regardless of baseline ECG findings [3].

From a technical standpoint, the models predominantly employed deep CNNs processing raw waveforms, a design that learns latent features rather than relying on pre-specified measurements. This distinguishes the included studies from traditional ECG risk scoring and explains their superior performance. Recent architectural innovations within this paradigm continue to yield significant gains: Lin et al. [12] (ECG-MACE) successfully deployed a multitask framework to predict multiple distinct outcomes simultaneously, while Sun et al. [10] demonstrated that coupling deep learning of raw waveforms with gradient boosting of clinical and demographic variables yields modest but consistent prognostic improvements. The shift towards survival neural network architectures (as seen in Tsai [17], Lin ECG-Surv [20], and Sau [23]) allows for sophisticated time-to-event modeling that vastly outperforms traditional binary classification.

External validation was performed in seven of thirteen studies, including transnational validation in Brazilian cohorts by Tsai et al. [17], which is encouraging for generalisability. However, six studies relied on internal validation alone, and performance attenuation in external cohorts was observed for some outcome-specific metrics — a pattern that warrants caution before assuming that headline AUC figures will replicate in new populations.

Three studies (Alrumayh et al. [22], Lu et al. [24] and Kanaji et al. [25]) employed a two-step approach in which the AI first identifies an intermediate phenotype — such as low ejection fraction, ECG-age gaps, or specific waveform abnormalities — before stratifying clinical outcomes. This methodology enhances interpretability by grounding predictions in recognisable pathophysiology, but introduces the risk of compounded prediction error and makes direct comparison with end-to-end prognostic models difficult.

When contextualizing these findings, the risk of bias within the included literature, as assessed via PROBAST, must be heavily weighed. While prospectively validated studies with robust methodological frameworks provide strong evidence, several included studies exhibited a high risk of bias—particularly due to retrospective designs, lack of independent external validation, or insufficient handling of missing data. The performance metrics reported by these high-risk models must therefore be interpreted with caution.

### 4.1 Clinical implications

The clinical case for AI-ECG prognostication rests on a combination of accessibility, additive value, and breadth of application. ECGs are ubiquitous, inexpensive, and collected routinely — deploying a prognostic layer on top of existing infrastructure requires no additional patient burden. The studies reviewed here demonstrate consistent incremental value over traditional clinical risk calculators (e.g., Lin ECG-MACE [12] outperforming the Framingham Risk Score; Hughes et al. [11] and Sau et al. [23] outperforming ASCVD Pooled Cohort Equations). This holds true even in populations with normal conventional ECG interpretations and preserved ejection fraction, where traditional tools provide little differentiation. Disease-specific application also appears highly viable: AI-ECG independently predicted 1-year mortality in HFrEF patients with a hazard ratio exceeding 4 (Lee et al. [21]), and successfully identified high-risk Takotsubo cardiomyopathy patients prone to recurrence and MACE (Kanaji et al. [25]). This suggests that these models can effectively stratify risk even within specialized populations that are already considered high-risk by conventional criteria. The incorporation of explainability techniques in some models — identifying the specific ECG regions driving predictions — is an important step toward clinical trust and regulatory acceptance. Collectively, these properties position AI-ECG as a scalable tool for population screening, perioperative triage, and risk stratification in resource-limited settings where advanced diagnostics are unavailable.

Finally, the integration of AI into clinical decision-making necessitates careful consideration of algorithmic transparency, the potential for embedded biases within training datasets, and patient data privacy, which remain ongoing ethical and regulatory challenges in the field [26].

### 4.2 Limitations

Several limitations of this review and the underlying evidence base should be acknowledged. Substantial outcome heterogeneity — in MACE definitions, follow-up durations, model objectives, and reported metrics — precluded meta-analytic pooling and limits direct cross-study comparisons. Six of the thirteen studies reported internal validation only, and performance attenuation in external cohorts, where reported, cautions against uncritical generalisation of headline figures. Twelve of the thirteen studies relied on standard 12-lead ECGs. While Alrumayh et al. [22] successfully demonstrated that AI analysis of a single-lead ECG can independently predict long-term clinical outcomes—providing a crucial proof-of-concept for smartwatch and wearable integration—the broader evidence base remains heavily skewed toward 12-lead clinical setups, limiting generalized conclusions about the prognostic utility of consumer wearables. Most studies did not report clinical utility metrics such as decision curve analysis or net reclassification improvement, which are essential for determining whether AI-ECG would actually change clinical management. Finally, several models were trained on proprietary or restricted datasets, limiting independent reproducibility.

Moreover, most studies did not report clinical utility metrics such as decision curve analysis (DCA) or net reclassification improvement. The absence of DCA is a critical barrier to clinical translation, as it leaves a gap in understanding the net clinical benefit of AI-ECG implementation across varying threshold probabilities—meaning we cannot yet definitively prove whether using these models safely alters clinical management and leads to better patient outcomes than current standard-of-care pathways.

Additionally, our search strategy was restricted to English-language publications, introducing a recognized language bias that may exclude relevant international cohorts.

Two further limitations merit acknowledgement. First, while discrimination (AUC/C-index) was universally reported, rigorous calibration assessment — the agreement between predicted and observed risk — was not universally prioritized across all studies. Although several of the larger, high-impact studies (such as Sun et al. [10], Ouyang et al. [18], and Sau et al. [23]) correctly reported calibration metrics and curves, other studies omitted this step. Because a model may discriminate well yet systematically over- or underestimate absolute risk in practice (as demonstrated by Champetier et al.‘s [19] finding of systematic overprediction in an external cohort), uniform reporting of calibration remains essential for safe clinical deployment. Second, the evidence base is geographically concentrated in high-income settings, with the majority of studies conducted in the United States, Taiwan, and the United Kingdom; the generalisability of these models to ethnically diverse and resource-limited populations — where AI-ECG could have the greatest impact — remains largely untested.

## 5. Conclusion

AI-enhanced ECG models represent a meaningful advance in cardiovascular risk prediction, demonstrating consistent moderate-to-excellent discrimination for all-cause mortality, cardiovascular mortality, and MACE across 13 studies and over ~5.9 million patients. The central finding of this review is not simply that these models perform well in isolation, it is that deep learning unlocks prognostic information already present in the routine ECG that conventional interpretation systematically fails to access. Moreover, several studies demonstrated that AI-ECG successfully stratifies risk even in patients with ‘normal’ human-interpreted ECGs or structurally normal hearts on imaging. This highlights that the AI is not merely replicating human diagnosis, but detecting novel subclinical phenotypes relevant across acute, chronic, perioperative, and community settings alike.

Translating this performance into clinical benefit requires prospective validation, standardised outcome reporting, calibration assessment, and evidence that AI-ECG-guided decisions improve patient outcomes rather than merely reclassifying risk. These remain open questions. If they are answered rigorously, AI-ECG has the potential to fundamentally expand the prognostic utility of one of medicine’s most accessible and widely performed tests.

## Supporting information

S1 Appendix Database search strategies, PROBAST quality assessment rationale, and supplementary study selection details. (DOCX)

S1 Table Complete all-cause mortality data. (DOCX)

S2 Table Complete cardiovascular mortality data. (DOCX)

S3 Table Complete major adverse cardiovascular events (MACE) data. (DOCX)

S1 Checklist PRISMA 2020 Checklist. (DOCX)
