# Supplementary material for: Artificial intelligence-enhanced electrocardiography for predicting mortality and major adverse cardiovascular events: A systematic review and narrative synthesis
Source: PLoS One. 2026 Oct 16;21(10):e0360756. doi: 10.1371/journal.pone.0360756 (PMC13674701; doi:10.1371/journal.pone.0360756)
Supplement: Supplementary file 1 [file pone.0360756.s001.docx]

# Supplementary appendix to: artificial intelligence-enhanced electrocardiography for predicting mortality and major adverse cardiovascular events: a systematic review and narrative synthesis

# Data base search strategies

Searches were conducted in four databases with the following date-specific strategies. All searches were restricted to English-language, peer-reviewed studies with no date limits applied.

Search conducted: November 25, 2025

## 1. PubMed/MEDLINE

("electrocardiogram"[Title/Abstract] OR "ECG"[Title/Abstract] OR "EKG"[Title/Abstract])

AND

("artificial intelligence"[Title/Abstract] OR "machine learning"[Title/Abstract] OR "deep learning"[Title/Abstract] OR "neural network*"[Title/Abstract])

AND

("cardiovascular mortality"[Title/Abstract] OR "cardiac death"[Title/Abstract] OR "mortality"[Title/Abstract] OR "major adverse cardiovascular event"[Title/Abstract] OR "MACE"[Title/Abstract])

NOT

("heart age"[Title/Abstract] OR "ECG age"[Title/Abstract])

## 2. IEEE Xplore

Advanced Search using Metadata Only (Title, Abstract, Index Terms):

("ECG" OR "electrocardiogram" OR "EKG")

AND

("artificial intelligence" OR "machine learning" OR "deep learning" OR "neural network")

AND

("mortality" OR "cardiovascular death" OR "MACE" OR "major adverse cardiovascular events")

AND NOT

("ECG age" OR "heart age")

*Filters: Peer-reviewed journals and conferences only*

## 3. Cochrane CENTRAL

Search strategy in title, abstract, or keywords:

## (ECG OR electrocardiogram OR EKG)

## AND

## (artificial intelligence OR machine learning OR deep learning OR "neural network*")

## AND

## (mortality OR "cardiovascular mortality" OR MACE OR "major adverse cardiovascular event*")

## NOT

## ("heart age" OR "ECG age")

## 4. Google Scholar

Due to the platform’s limited advanced search syntax, a simplified string was used, and the first 200 relevance-sorted results were manually screened. Search string:

"artificial intelligence"

AND

("ECG" OR "electrocardiogram")

AND

("mortality" OR "MACE") -"ECG age" -"heart age"

Note: Results were manually screened for relevance due to Google Scholar's limited advanced search capabilities. Only the first 200 results were screened.

**Supplementary Study Selection Details**

Our systematic search identified 514 records across four databases: PubMed/MEDLINE (n=37), IEEE Xplore (n=207), Cochrane CENTRAL (n=70), and Google Scholar (n=200 manually screened). After removing 2 duplicates, 512 records were screened by title and abstract, resulting in 485 exclusions. Twenty-seven full-text articles were assessed for eligibility, of which 14 were excluded due to incompatible focus (ECG-age estimation, n=4; diagnostic-only outcomes, n=5; pediatric population, n=1; Conference Abstract, n=1; Non-AI/non-deep learning model n=3).

Thirteen studies met all inclusion criteria and were included in the final qualitative synthesis.

The PRISMA flow diagram (Fig 1) provides a detailed account of the screening process.

**Quality assessment of included studies**

We assessed the methodological quality and risk of bias of included studies using the Prediction model Risk Of Bias ASsessment Tool (PROBAST)^14^, which evaluates four domains: participants, predictors, outcome, and analysis. PROBAST is specifically designed for systematic reviews of prognostic model studies and provides a structured framework for evaluating both risk of bias and concerns regarding applicability.

Assessment Methods

Two reviewers independently assessed each study across the following domains:

1. Participants: Appropriate data sources, inclusion/exclusion criteria, and participant representativeness

2. Predictors: Objective measurement and availability of ECG data at the time of prediction

3. Outcome: Clear outcome definition, appropriate measurement methods, and adequate follow-up

4. Analysis: Adequate sample size, appropriate handling of missing data, model validation, and reporting of performance metrics

For each domain, risk of bias was categorized as Low, High, or Unclear. Overall risk of bias was judged as Low only if all domains were rated as Low risk.

Detailed Findings by Domain

Domain 1: Participants

Ten of the thirteen studies (77%) were rated as low risk for the participants domain. Most studies used large, well-defined cohorts from established healthcare databases or registries with clear inclusion and exclusion criteria. Three studies received unclear ratings: Ouyang et al. due to a preoperative patient selection that may limit generalisability to broader general medical populations; Kanaji et al. due to a highly selective population of Takotsubo cardiomyopathy patients at a single centre; and Alrumayh et al. due to recruitment from patients already attending for routine echocardiography, which introduces potential referral bias.

Domain 2: Predictors

All thirteen studies (100%) were rated as low risk for the predictors domain. All studies used standardised 12-lead or single-lead surface ECGs obtained through routine clinical practice. ECG data were objective, digitally recorded, and available at the time prediction would be made clinically, ensuring predictor reliability and clinical applicability.

Domain 3: Outcome

All thirteen studies (100%) were rated as low risk for outcome assessment. Mortality outcomes were ascertained through linkage to national death registries or electronic health records, ensuring objective and complete outcome data. MACE outcomes were defined using standardised ICD codes or validated registry data.

Domain 4: Analysis

Nine of the thirteen studies (69%) were rated as low risk for analysis methods. These studies used appropriate sample sizes, applied proper statistical methods, performed internal or external validation, and adequately reported model performance metrics. Two studies received an unclear rating: Lu et al., due to a non-standard two-step predictor handling design in which AI-identified ECG phenotypes serve as intermediate predictors rather than direct model outputs, raising uncertainty regarding signalling question 4.2; and Lee et al., due to small dataset size and reliance on internal validation alone. Two studies received high risk ratings: Kanaji et al. due to a small sample of 305 patients with limited events for multivariable survival analysis and absence of external validation; and Alrumayh et al. due to internal validation only within a single prospective cohort and limited reporting of missing data handling.

Overall Risk of Bias and Applicability

Eight studies (62%) demonstrated low overall risk of bias across all four domains, indicating high methodological quality. Three studies (23%) had unclear overall risk: Ouyang et al. due to a selective preoperative patient population; Lee et al. due to small dataset size and internal validation only; and Lu et al. due to non-standard predictor handling in the analysis domain. Two studies (15%) had high overall risk, attributable to small sample sizes or restricted validation (Kanaji, Alrumayh).

Regarding applicability, seven studies (54%) had low concerns, indicating their findings are broadly applicable to general clinical populations. Five studies (38%) had unclear applicability concerns: Raghunath et al. due to derivation from a single US health system with limited geographic and demographic diversity; Ouyang et al. and Champetier et al. due to perioperative patient selection; Lee et al. due to restriction to HFrEF patients; and Alrumayh et al. due to recruitment from a referral echocardiography population. One study (8%) had high applicability concerns due to its highly specialised population (Takotsubo cardiomyopathy; Kanaji et al.).

**Summary:**

The majority of included studies demonstrated rigorous methodology with low risk of bias, particularly in the domains of predictors and outcomes, where all thirteen studies were rated as low risk. The use of standardised, objective ECG data and validated outcome measures strengthens the reliability of the findings across the review. The primary methodological limitations identified were concentrated in the analysis domain, where two studies — Kanaji et al. and Alrumayh et al. — received high risk ratings due to small or single-cohort validation designs, and one further study — Lu et al. — received an unclear rating due to its two-step predictor handling approach. Applicability concerns were present in a minority of studies, reflecting the inherent trade-off between specialised populations yielding clinically meaningful findings in specific contexts and the generalisability of those findings to broader practice. These quality assessments were considered in the interpretation of results and inform our recommendations for future research prioritising larger, externally validated, prospective studies across diverse clinical settings.
