# Supplementary material for: Artificial intelligence-enhanced electrocardiography for predicting mortality and major adverse cardiovascular events: A systematic review and narrative synthesis
Source: PLoS One. 2026 Oct 16;21(10):e0360756. doi: 10.1371/journal.pone.0360756 (PMC13674701; doi:10.1371/journal.pone.0360756)
Supplement: Supplementary file 2 [file pone.0360756.s002.docx]

**S1 Table. Complete All-Cause Mortality Data**

| **Study** | **Timepoint** | **Sample Size** | **AUC/C-index (95% CI)** | **HR (95% CI)** | **Validation** |
| --- | --- | --- | --- | --- | --- |
| Raghunath (2020) | 1-year | 168,914 ECGs | 0.88 | 9.5 | Internal |
| Sun (2023) | 30-day | 97,144 ECGs | 0.843 (0.838–0.848) | NR | Internal |
|  | 1-year | 89,379 ECGs | 0.812 (0.808–0.816) | NR |  |
|  | 5-year | 55,650 ECGs | 0.798 (0.792–0.803) | NR |  |
| Lin ECG-surv (2025) | 1-year | 4,900,000 ECGs | 0.860 (0.859–0.861) internal / 0.813 (0.807–0.814) external | NR | Both |
| Lin ECG-MACE (2025) | 1-year (internal) | 2,800,000 ECGs | 0.89 | NR | Both |
|  | 1-year(external) |  | 0.83 |  |  |
| Ouyang (2024) | 30-day | 4,581 pts | 0.83 (0.79–0.87) | NR | Both |
|  | 30-day (external - CUMC) | 9,028 pts | 0.79 (0.75–0.83) |  |  |
|  | 30-day (external - SHC) | 101,375 pts | 0.75 (0.74–0.76) |  |  |
| Champetier (2025) | 30-day | 6,098 patients | 0.707 (0.672–0.742) | NR | External |
| Sau (2024)* | Mean 5.46yr (internal — BIDMC) | 1,163,401 ECGs | 0.775 (0.773–0.776) | NR | Both |
|  | Mean 5.46yr (external — UKB) |  | 0.638 (0.608–0.668) | NR |  |
| Lee (2025) | 1-year | 3,894 patients | 0.826 (0.794-0.859) | 4.12 (2.32–7.33) | Internal |
| Lu (2024) | 7-year | ~1,600,000 patients | NR | ST: 2.24 (1.96–2.57); AF: 2.22 (1.99–2.48) | Internal |
| Alrumayh (2025) | 2-year | 1,007 patients | 0.75 | 1.56 (1.06–2.29) | Internal |
| Tsai (2023)** | 1-year (internal) | 512,805 ECGs | 0.894 (0.879–0.909) | NR | Both |
|  | 1-year (external — Hospital B) |  | 0.858 (0.844–0.872) | NR |  |
|  | 10-year (internal) |  | 0.858 (0.854–0.862) | 14.16 (11.33–17.70) |  |
|  | 10-year (external — Hospital B) |  | 0.836 (0.832–0.841) | 10.59 (8.32–13.48) |  |

*Abbreviations: AF, atrial fibrillation; ST: sinus tachycardia; AUC, area under the curve; CI, confidence interval; HR, hazard ratio; NR, not reported.*

**Sau et al. additionally reported c-indices of 0.762 (CODE-10s), 0.773 (SaMi-Trop), and 0.713 (ELSA-Brasil) across external cohorts using model variants fine-tuned for primary care settings.*

*** Tsai et al. additionally validated in two transnational cohorts: SaMi-Trop (Brazil, Chagas cardiomyopathy, n=1,631) HR 4.91 (2.63–9.16); CODE15 (Brazil, n=233,647) HR 2.29 (2.15–2.44)*
