# Supplementary material for: Artificial intelligence-enhanced electrocardiography for predicting mortality and major adverse cardiovascular events: A systematic review and narrative synthesis
Source: PLoS One. 2026 Oct 16;21(10):e0360756. doi: 10.1371/journal.pone.0360756 (PMC13674701; doi:10.1371/journal.pone.0360756)
Supplement: Supplementary file 3 [file pone.0360756.s003.docx]

**S2 Table. Complete Cardiovascular Mortality Data**

| **Study** | **Timepoint** | **Sample Size** | **AUC/C-index (95% CI)** | **HR (95% CI)** | **Validation** |
| --- | --- | --- | --- | --- | --- |
| Hughes (2023) | 5-year (internal) | 31,899 pts | 0.832 (0.810–0.854) | NR | Both |
|  | 5-year (external-Cedars-Sinai) |  | 0.781 (0.767–0.795) |  |  |
|  | 5-year (external — Columbia) |  | 0.825 (0.821–0.829) |  |  |
| Lin ECG-surv (2025) | 1-year | 4,900,000 ECGs | 0.891 (0.890–0.893) | NR | Internal |
| Sau (2024) | Mean 5.46yr (internal — BIDMC) | 1,163,401 ECGs | 0.832 (0.831–0.834) | NR | Both |
|  | Mean 5.46yr (external — UKB) |  | 0.695 (0.636–0.754) |  |  |
| Tsai (2023) | 10-year | 512,805 ECGs | NR | 18.50 (9.82–34.84) | Internal |
