# Supplementary material for: Artificial intelligence-enhanced electrocardiography for predicting mortality and major adverse cardiovascular events: A systematic review and narrative synthesis
Source: PLoS One. 2026 Oct 16;21(10):e0360756. doi: 10.1371/journal.pone.0360756 (PMC13674701; doi:10.1371/journal.pone.0360756)
Supplement: Supplementary file 4 [file pone.0360756.s004.docx]

**S3 Table. Complete Major Adverse Cardiovascular Events (MACE) Data**

**A. Composite MACE Outcomes**

| **Study** | **MACE Definition** | **Timepoint** | **Sample** | **AUC (95% CI)** | **HR (95% CI)** | **Validation** |
| --- | --- | --- | --- | --- | --- | --- |
| Ouyang (2024) | Mortality, cardiac arrest, MI, heart block, pulmonary oedema | 30-day | 45,969 pts | 0.77 (0.73–0.80) | NR | Internal |
| Champetier (2025) | AMI, acute HF, life-threatening arrhythmia, death | 30-day | 6,098 pts | 0.675 (0.649–0.701) | NR | External |
| Kanaji (2024) | CV death, MI, HF hospitalisation, stroke, Takotsubo recurrence | Median 4.8y | 305 pts | NR | 4.42 (1.83–10.66) | Internal |
| Alrumayh (2025) | All-cause mortality, acute MI, HF hospitalisation, stroke/TIA | 2-year | 1,007 pts | 0.75 | 1.93 (1.39–2.69) | Internal |

**B. Individual MACE Components**

| **Study** | **Outcome** | **Timepoint** | **Sample** | **AUC (95% CI)** | **HR (95% CI)** | **Validation** |
| --- | --- | --- | --- | --- | --- | --- |
| Lin ECG-MACE (2025) | HF hospitalization | 1-year | 2.8M ECGs | 0.90 | NR | Internal |
|  | MI | 1-year |  | 0.85 | NR |  |
|  | Stroke | 1-year |  | 0.76 | NR |  |
| Tsai (2023) | AMI | 10-year | 512,805 ECGs | NR | 4.01 (2.24–7.17) | Internal |
|  | Heart failure | 10-year |  | NR | 6.66 (4.54–9.77) |  |
|  | Stroke | 10-year |  | NR | 2.15 (1.70–2.72) |  |
| Hughes (2023) | MI | 5-year | 31,899 pts | NR | 2.0 (1.7–2.5) | Internal |
|  | HF hospitalization |  |  | 0.76 (0.74–0.77) | 3.2 (2.9–3.5) | Internal |
|  | Stroke |  |  | NR | 1.6 (1.4–1.9) | Internal |
|  | SCD |  |  | NR | 4.2 (3.0–5.7) | Internal |
|  | ASCVD (composite) |  |  | 0.67 / 0.63 | 2.4 (2.2–2.7) | Both |
| Sau (2024) | ASCVD (composite) | Mean 5.46yr | 1,163,401 ECGs | 0.696 (0.694–0.698) internal / 0.643 (0.624–0.662) external | NR | Both |
|  | HF hospitalization |  |  | 0.787 (0.785–0.789) internal / 0.768 (0.733–0.802) external |  |  |
|  | Ventricular arrhythmia |  |  | 0.760 (0.756–0.763) internal / 0.719 (0.635–0.803) external |  |  |

*Abbreviations: aHR, adjusted hazard ratio; AMI, acute myocardial infarction; AUC, area under the curve; CI, confidence interval; HF, heart failure; HR, hazard ratio; MACE, major adverse cardiovascular events; MI, myocardial infarction; SCD, sudden cardiac death; NR, not reported; pts, patients.*
